# Determination of the Major By-Products of *Citrus hystrix* Peel and Their Characteristics in the Context of Utilization in the Industry

**DOI:** 10.3390/molecules28062596

**Published:** 2023-03-13

**Authors:** Martyna Lubinska-Szczygeł, Anna Kuczyńska-Łażewska, Małgorzata Rutkowska, Żaneta Polkowska, Elena Katrich, Shela Gorinstein

**Affiliations:** 1 Department of Analytical Chemistry, Faculty of Chemistry, Gdańsk University of Technology, 80-233 Gdańsk, Poland; malgorzata.rutkowska@pg.edu.pl; 2 Department of Energy Conversion and Storage, Faculty of Chemistry, Gdańsk University of Technology, 80-233 Gdańsk, Poland; anna.lazewska@pg.edu.pl; 3 Institute for Drug Research, School of Pharmacy, Faculty of Medicine, The Hebrew University of Jerusalem, Jerusalem 9112001, Israel; ekatrich@gmail.com (E.K.);

**Keywords:** kaffir lime, by-products, essential oil, pectins, bioactive compounds, antioxidant activity, thermodynamic properties, minerals

## Abstract

Kaffir lime (*Citrus hystrix*) is a popular citrus in Southeast Asia. Despite the growing interest in the peel of the fruit, the leaves are the most frequently used part of the fruit. The aim of the study was to determine the main by-products of the peel, such as pectins, minerals, essential oil, and bioactive compounds, and to evaluate the possibility of using them in various branches of industry. In the study of the essential oil obtained by hydrodistillation performed using the TGA chromatography technique (GC-MS), sabinene (31.93%), β-pinene (26%), and limonene (19%) were selected as the most abundant volatile compounds. Nine microelements (Fe, Zn, Cu, Mn, Co, Ni, Cr, Mo, and V), four macroelements (Mg, Ca, K, and Na), and seven ballast substances (Cd, Hg, Pb, Al, V, Sr, and Pt) were also determined using the microwave plasma-atomic emission spectrometry technique (MP-AES). In the case of microelements, iron 32.72 ± 0.39 mg/kg DW (dry weight) had the highest concentration. In the case of macroelements, the calcium content was 9416 ± 34 mg/kg DW. Optimization of the pectin extraction was also performed by selecting citric acid and obtaining a yield of 7.6–17.6% for acid extraction and 9.9–28.2% for ultrasound-assisted extraction (UAE), depending on the temperature used. The obtained pectins were characterized by the degree of methylation, galacturonic acid content, 2,2-diphenyl-1-picrylhydrazyl (DPPH) free radical scavenging, and DSC (differential scanning calorimetry) analysis. Among bioactive compounds, the contents of polyphenols (22.63 ± 2.12 mg GAE/g DW), flavonoids (2.72 ± 0.25 mg CE/g DW, vitamin C (2.43 ± 0.19 mg Asc), xantoproteins + carotenes (53.8 ± 4.24 ug), anthocyanins (24.8 ± 1.8 mg CGE/kg DW), and chlorophylls A and B (188.5 ± 8.1, 60.4 ± 3.23 µg/g DW) were evaluated. Antioxidant capacity using (cupric ion-reducing antioxidant capacity) CUPRAC and DPPH assays was also provided with the results of 76.98 ± 8.1, and 12.01 ± 1.02 µmol TE/g DW, respectively.

## 1. Introduction

Citrus fruits belong to the group of the largest crops in the world, with an annual production of more than 146 million tons [1]. The largest world citrus producers are China, Brazil, the USA, India, and Mexico [2]. Almost 33% of crops, especially oranges, are industrially processed for juice production. However, more than half of the processed citruses, including peel, are citrus waste, a possible source of environmental pollution [3]. The problem is not only post-production waste, but also fruits discarded for commercial reasons and fruit discarded because of regulations that limit production [4]. The main ways to use citrus waste are the production of biofuels, in particular, ethanol and biogas; in essential oils; or in the production of cattle feed [5,6]. Valorization of citrus peels for the recovery of by-products seems to be economically and environmentally reasonable. Despite that there are some limitations of citrus peels in industrial applications, such as instability, poor water solubility, and low bioavailability, the future trends seem to be promising [7].

There are also fruits in which pulp and juice are not directly consumed because their taste is too pungent. An example of such a fruit is kaffir lime (*Citrus hystrix*), popular in Asian countries. The leaves are the most frequently used part of the plant in the context of culinary purposes [8]. The outer peel is used to make curry paste. Thanks to the pleasant sensory properties and a large amount of fruit remaining after collecting leaves, kaffir lime fruits can often be found in toilets, where they are used as air fresheners. The candied kaffir lime peel can be found in Cambodia, but it is not very popular. In addition to its pleasant sensory properties, dried kaffir lime peel is an excellent additive to cakes or desserts. Kaffir lime has been used in traditional medicine for treating various illnesses, for example, cold pain [9]. This is confirmed in the literature, where the potential therapeutic effects of *Citrus hystrix* DC on metabolic disorders have been demonstrated [10]. Kaffir lime essential oils are utilized in the production of cosmetics [11]. According to recent literature reports, kaffir lime essential oil peel is safe for the skin in an in vitro model [12]. There are also many articles about the characteristics and use of the leaves of this fruit [13]. However, the research area on kaffir lime peel still needs to be completed.

The inner part of the citrus peel is also a rich source of pectins, which constitute about 30% of its content. Although pectins were discovered more than 200 years ago, their physicochemical and structural properties are still under investigation owing to the great diversity of this family of polymers and the close relationship between structure and function. More and more research is being carried out because of the new sources and conditions of pectin extraction. Plant raw materials are increasingly used for pectin extraction. The pectin content and their characteristics depend on the plant species from which the pectin is isolated and the plants’ age or degree of maturation. Lemon peel is particularly rich in pectin, from which 36.71% was extracted [14]. Owing to the thick albedo, good pectin yields have also been obtained from pomelos [15]. Thick, wrinkled kaffir lime’s peel appears to be a very promising source of pectin. The composition of pectins is strongly related to their source, extraction, and purification method [14]. Pectins for specific applications are selected depending on their composition and structure (percentage of galacturonides and neutral sugars, degree of esterification, and so on). According to reports, polysaccharides from plants have been considered as a novel potential antioxidant source thanks to their low toxicity and high level of antioxidant capacity, such as their radical scavenging abilities [16]. DPPH radical scavenging assay has been widely used for the determination of the antioxidant activity of pectins [17]. An extremely important aspect of determining pectin properties is also their thermodynamic characteristics, which affect their quality in the final application. Considering the importance of the structural and physical properties in the functionality of pectins, it is important to provide their characterization.

Citrus peels are also good sources of bioactive compounds, such as polyphenols, flavonoids, anthocyanins, or vitamins, showing many properties [18]. Earlier literature reports showed that kaffir lime peel contains a similar polyphenol content to the most commonly consumed orange peel [8,19]. These attributes are commonly used while producing new dietary supplements, cosmetics, or medicines containing plant ingredients. Another important group of compounds present in citrus waste are micro- and macroelements. Although there are no processes aimed at obtaining minerals from citrus waste, the knowledge of their content is important in the context of using citrus waste as animal feed. All these compounds are necessary for the proper development of organisms and are involved in many metabolic processes. According to the best of our knowledge, there is a lack of scientific reports about the content of micro- and macroelements of kaffir lime peel and the state of knowledge of bioactive compounds of kaffir lime has not been exhausted yet.

In this study, major functional components from the by-products of kaffir lime peels, such as essential oils, pectins, micro- and macroelements, and bioactive compounds (polyphenols, flavonoids, vitamin C, anthocyanins, chlorophyll A and B, carotenoids (xanthophylls + carotenes)), were evaluated and characterized. Determination of antioxidant activity FRAP and CUPRAC was carried out. The results provide a full characteristic of the peel of kaffir lime. It is the background for potential industrial applications. Getting to know the health benefits of consuming kaffir lime peel products could contribute to the spread of this fruit, not only in Asian countries. The insight provided could be useful in food science and technology.

## 2. Results and Discussion

### 2.1. Essential Oil

#### 2.1.1. Essential Oil Extraction Efficiency

Hydrodistillation is one of the most simple and still the most common methods for the extraction of essential oil (EO) from plant materials. It allows obtaining a relatively high yield of obtained oil with the use of an eco-friendly solvent. Though raw materials are directly immersed in boiling water, the water acts as a protective barrier and prevents the extracted essential oil from overheating to a certain extent [11]. In earlier studies that used the hydrodistillation technique to obtain essential oils from fresh kaffir lime peel, the yield was 0.16–2.10% [20,21]. On the other hand, in our research, 1.78% of the oil was obtained, which is a very good result compared with previous studies. Hongratanaworakit et al. obtained 1.5% of the essential oil by distilling it for 2 h [22]. However, it should be remembered that the efficiency of obtaining essential oils depends not only on the conditions of the extraction process, but also on the harvesting regime, drying mode, and storage period [23]. Among the Citrus species, *C. sinensis* exhibited the maximum oil yield (0.24–1.07%) followed by *C. reticulata* (0.30–0.50%) and *C. paradisi* (0.20–0.40%). So, the kaffir lime is a promising source of essential oil production in terms of the amounts of oil extracted.

#### 2.1.2. Essential Oil Composition

The second important feature when selecting raw materials for obtaining EOs is their composition. Citrus fruits’ EOs consist mostly of terpenes, which have several bioactive properties, as well as pleasant aromas, thanks to which the oils are widely used in the pharmaceutical and cosmetic industries [24]. Depending on the terpene profile, essential oils have specific health-promoting and aromatic properties, which is a key element in the stage of designing new drugs and cosmetics. The use of kaffir lime oil in the production of cosmetics has become more and more popular in recent years [11].

Some research on kaffir lime oil composition has been provided so far [25]. In most cases, β-pinene, limonene, and sabinene were major chemical compounds [26,27]. It should be remembered that the content of volatile substances in essential oils depends on the geographical origin of the fruit [25]. Warsito et al. determined citronellal as the major component of *Citrus hystrix* essential oils, with small amounts of terpenes described above [28].

The results of the study of the terpene kaffir lime peel oil profile are shown in Table 1. The most abundant terpene was sabinene, with a percentage of 31.9%. This chemical compound, with a woody aroma, has anti-inflammatory, antioxidant, antiviral, anti-diabetic, and anticancer properties [29], which is an important element for the use of oil for industrial purposes. The contents of β-pinene with a herbal aroma (26.3%) and limonene with a citrus aroma (18.6%) are also very high. These three chemicals play a key role in creating the aroma of kaffir lime oil, which is described in the cosmetic industry as fresh and sharp.

**Figure 1:**
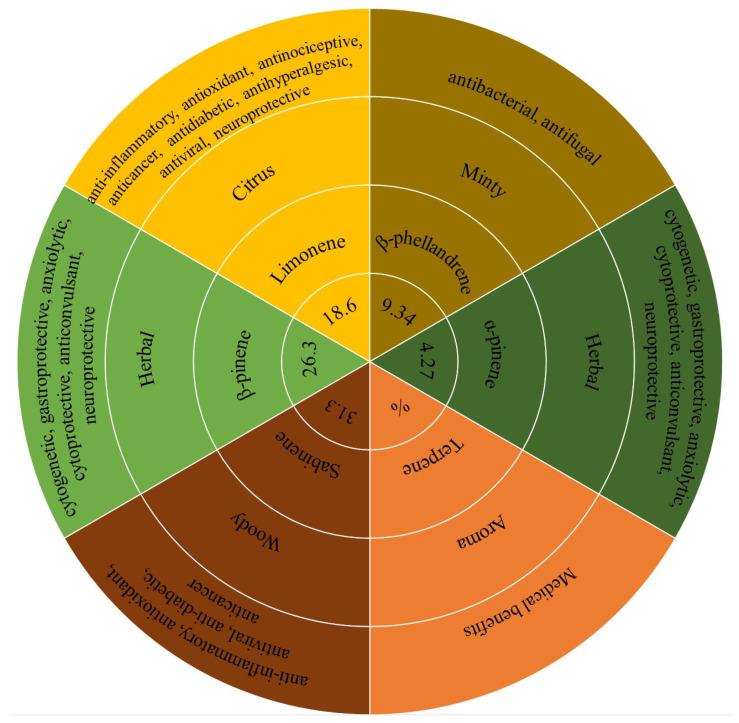
Terpene profile chart of kaffir lime essential oil.

Other important compounds making up the composition of kaffir lime essential oil are β-phellandrene, α-pinene, myrcene, and p-cymene, belonging to the group of monoterpene hydrocarbons. The results are consistent with the results obtained by Baccati et al. [30], although with a slight predominance of sabinene over β-pinene in our case. As already mentioned, the difference may be due to the geographical origin of the fruit, the degree of ripeness, or the method of extracting essential oils. The content of many terpenes with several bioactive properties makes kaffir lime oil a mixture with many health benefits (Figure 1).

Proven antibacterial and antifungal properties lead to the use of kaffir lime oils in the production of oral sprays, mouthwashes, and acne-control cosmetics [11]. Their use in aromatherapy is also common [11]. Our research is consistent with previous literature reports.

### 2.2. Pectins

#### 2.2.1. Optimization of Pectin’s Extraction Conditions

##### Selection of Temperature and Type of Acid

**Figure 2:**
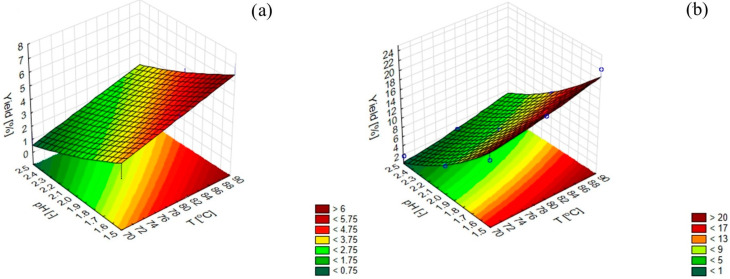
The efficiency of pectin extraction from kaffir lime depending on the extraction conditions for (**a**) nitric acid and (**b**) citric acid.

The figure shows the efficiency of pectin extraction from kaffir lime depending on the extraction conditions and the type of acid used (Figure 2). Based on the conducted research, it can be concluded that the selection of citric acid to obtain the set pH value during pectin extraction seems to be a good solution, as it allows for a very good product yield—22%. When nitric acid was used, an extraction yield of less than 8% was obtained. The results are consistent with those obtained by Shaha et al., where the best efficiency of pectin extraction from microwave-dried kaffir lime was obtained for citric acid [31]. In the case of temperature, along with its increase, the efficiency of pectin extraction increased. A temperature of 90 °C was used as a limit variable in the design of the experiment. In the further part of the experiment, it was not decided to increase the temperature to 90 °C because of the possible degradation of the obtained pectins [32]. Instead, pectins extracted at 60 °C were analyzed.

##### Determination of the Influence of Ultrasound on the Extraction Efficiency

As mentioned above, in further studies, the extraction temperature was lowered in order not to degrade pectins. Based on the above results, citric acid was selected for extraction.

Pectin yield is one of the most important parameters taken into account while choosing the extraction conditions. The yields of pectins obtained using different extraction conditions are presented in Table 2. Based on the literature research and experiments conducted, citric acid was chosen as an extracting agent, being a better option than nitric acid [31]. In addition, by raising the extraction temperature to 80 °C, a yield of about 18% was obtained. The use of ultrasound enables extractions at lower temperatures, ensuring a higher yield and better quality, but may cause a difference in their chemical structures [33]. In the case of UAE when heated to 60 °C, a yield of 10% was obtained and raising the temperature to 80% allowed to increase the efficiency of this method more than three times. Rodsamran et al. proved a better pectin yield of lime for the conventional method of pectin heating for a higher peel-to-extractant ratio (1:40) for citric acid. The value was 19.63%. Therefore, it seems reasonable to use a high ratio of dried peel to extrahent like in our case (1:50) for citric acid. Sayah et al. studied the yield of pectin from grapefruits and oranges, obtaining results at the level of 22.69–33.69% [34]. In turn, the pectin content of *Citrus reticulata* using subcritical water was determined in a quantity of 19.21–21.95% [35]. Therefore, comparing the pectin content of kaffir lime peel to other citruses, this fruit seems to be a very good source of these chemical compounds.

#### 2.2.2. Degree of Methylation

The functionality of pectins in food products largely depends on their degree of methylation and polymerization. High methoxylpectins form a gel at acidic pH (approximately 3) in the presence of a high sugar concentration (approximately 65%), while low methoxylpectins require divalent ions to form gels, but can be used over a wider pH range (3–6) and with a lower sugar content (30–40%). Therefore, owing to dietary restrictions, low methyl pectins are more commonly used in the food industry [36].

The degree of pectin methylation determined by the titration method is presented in the table (Table 3). Based on the results of the analysis, it can be stated that the extraction conditions did not have a significant effect on the degree of esterification of the obtained pectins. In all cases, the degree of esterification was 3–4%. This means that the pectins extracted from kaffir lime belong to the group of low methoxylpectins and would be a good option for the food industry.

#### 2.2.3. Galacturonic Acid Content

Table 4 shows the content of galacturonic acid in the samples of kaffir lime depending on the extraction conditions used. The highest content was recorded in the case of using acid extraction and a temperature equal to 60 °C. As the temperature increased, the content of galacturonic acid in the samples of kaffir lime decreased. Moreover, it was noticed that supporting the extraction with ultrasound also decreased the content of the determined chemical compound. According to the regulations, pectins used in the food industry should contain a minimum of 65% of uronic acid [37], so only the use of a temperature of 60 °C made it possible to meet this criterion. For comparison, orange peel pectins contained 422 mg/g and 1099 mg/g of uronic acid, depending on the extraction method used [37].

#### 2.2.4. DPPH Free Radical Scavenging

**Figure 3:**
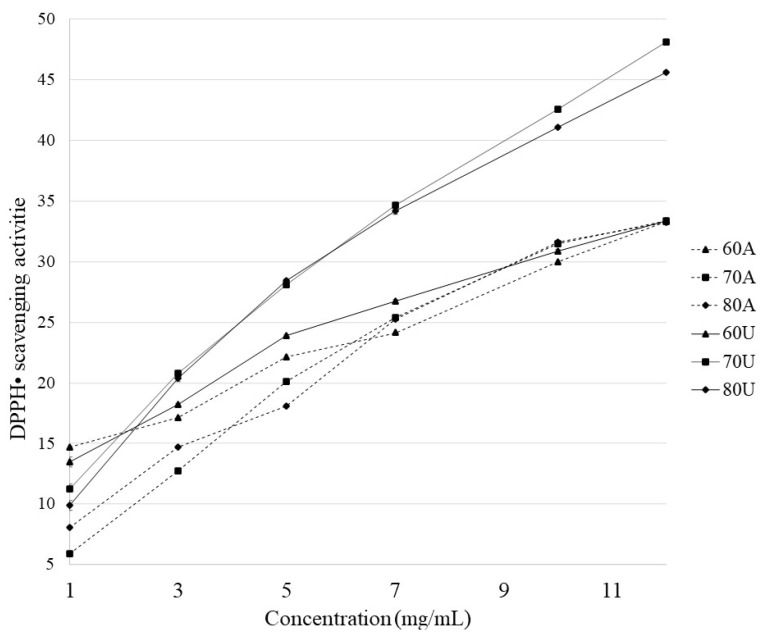
DPPH free radical scavenging of pectins extracted from kaffir lime.

DPPH free radical scavenging is a mechanism commonly used for screening the antioxidant activity of plant extracts [38]. The results of DPPH free radical scavenging of pectins extracted from kaffir lime are presented in Figure 3. The antioxidant activities for the extracted pectins with two different methods were increased in a concentration-dependent manner from 1 to 12 mg/mL. It is noticed that there are no several differences between the impact of the temperature of pectin extraction by the conventional method for the antioxidant potential of pectins obtained. However, there was an increase in DPPH• scavenging activity for pectins extracted using ultrasounds at the same temperatures as the conventional method. This is in agreement with previous literature reports that using ultrasound-assisted methods of extraction of pectins affects larger values of antioxidant potential of pectins (higher DPPH• scavenging activity) [39]. Compared with data provided by Gharibzahedi et al., the results for vitamin C at a concentration of 12 mg/mL were much higher (about 85%). On the other hand, in the reported research [40] for solutions of pectin samples from figs with a concentration of 12 mg/mL, a DPPH• scavenging rate of about 60% was obtained. Compared with our results, kaffir lime peel pectins show lower values of these parameters: 33.25 ± 0.17% (80A), 48.13 ± 0.15% (70U), which proves relatively low antioxidant activity. It is thus not suitable for use in biomedical applications. Low antioxidant activity may also indicate the high viscosity of extracted pectins [40]. In turn, that proves the good gelling properties and the possibility of use in the food industry.

#### 2.2.5. DSC Analysis

In drug development, preformulation plays a key role and provides much information about the product. DSC thermal study is one of the preferred techniques because it provides general information about the thermal transition as well as chemical stability and properties.

The effects of extraction temperature and the addition of ultrasounds in the extraction process on the thermodynamic properties of pectin were examined by DSC between 30 °C and 300 °C. As shown in Figure 2, an endothermic peak and an exothermic peak were observed in the DSC thermograms of all pectin samples. The parameters of the two peaks are listed in Table 5, such as the maximum temperature of the main endothermic peak (T_endo_), enthalpy of the main endothermic peak (ΔH_endo_), degradation temperature (T_exo_), and degradation enthalpy (ΔH_exo_).

**Figure 4:**
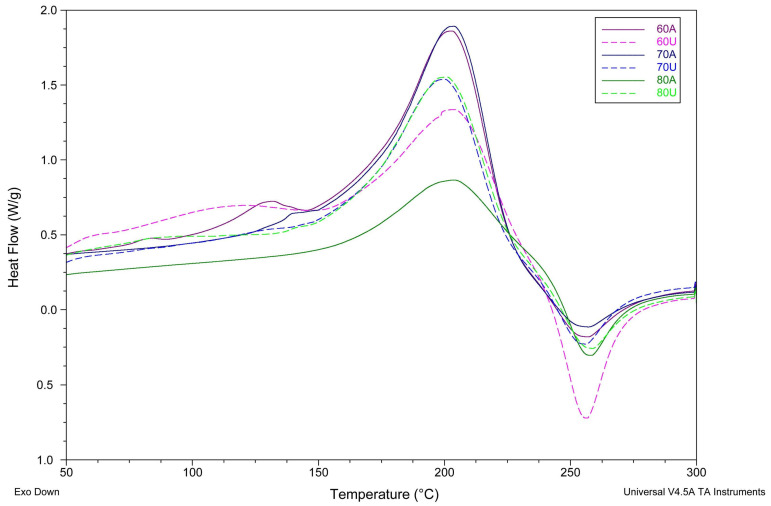
DSC thermograms in the full temperature range of citrus pectins extracted at 60 °C, 70 °C, and 80 °C by (A) citric acid in pH 1.5 and (U) citric acid in pH 1.5 with the addition of ultrasounds, precipitated using methanol.

Because the endothermic phenomenon is assigned to water evaporation [41], a higher melting temperature and melting enthalpy meant more energy was needed to absolutely remove water. There is no clearly visible trend associated with the enthalpy value. The endothermic peak starts above 90 °C, which is connected to water evaporation. In samples extracted at lower temperatures without the addition of ultrasounds, one lower peak (at 130 °C for 60A and 140 °C for 70A—Figure 4) can be seen. For these two samples (60A and 70A), enthalpy is similar and above 300 J/g. Samples after using ultrasound for the extraction have no additional peak and enthalpy is lower between 191.1 J/g and 276.5 J/g for 60U, 80U, and 70U, in order.

T_endo_ values ranged from 200.26 to 204.77 °C. The lowest temperature was observed for the sample extracted at 70 °C in citric acid with the addition of ultrasound (70U). The highest temperatures were obtained for the samples without additional ultrasound treatment (70A and 80A),with the exception of samples extracted at 60 °C (60A and 60U). For these samples, the temperature difference is smaller and the highest temperature of all samples was observed for the sample extracted with the addition of ultrasounds at 60 °C (60U).

**Figure 5:**
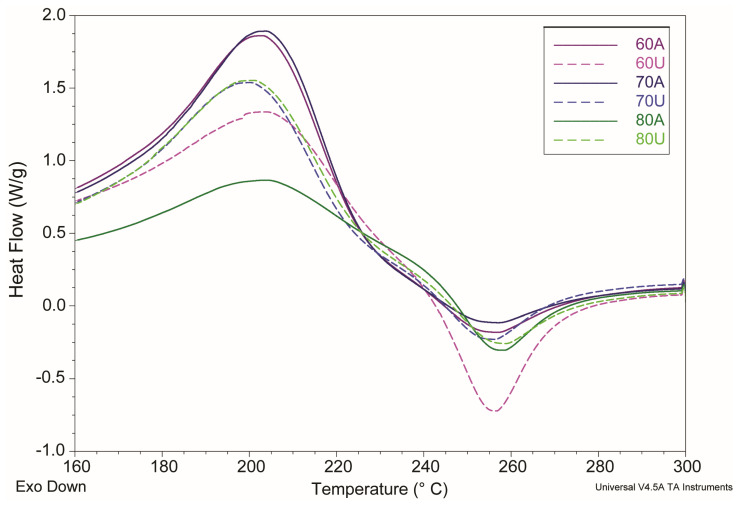
Fragments of DSC thermograms of citrus pectins extracted at 60 °C, 70 °C, and 80 °C by (A) citric acid in pH 1.5 and (U) citric acid in pH 1.5 with the addition of ultrasounds, precipitated using methanol, in the 160 °C–300 °C temperature range.

The second peak was caused by the degradation of pectin in heat processing [42]. As shown in Table 5, samples showed little differences for T_exo_ from 253.49 °C to 256.84 °C. The temperature T_exo_ range is similar to temperatures reported for exothermic peak for amidated pectins in the literature [41]. Amidated pectins have a lower amount of methoxyl groups, which is consistent with similarities between the literature results of DSC measurements for low-methoxylated pectins [43] and the results for pectin samples extracted for this publication (Figure 5).

In Figure 5, it can be noticed that peaks obtained for the pectins described in this paper were more separated than peaks for the low-methoxylated pectins prepared by acidic treatment (LMP-A) described in the literature by Einhorn-Stoll et al. in 2020 [43]. This means lower melting temperatures and a higher amount of water in extracted pectins.

A shift of the DSC curves to a lower temperature (earlier pyrolysis) is characteristic of pectin demethoxylation [41]; this shift was less prominent for samples extracted at higher temperatures or with the addition of ultrasounds (Figure 5). The most prominent shift was observed for the sample 60U, which was extracted at 60 °C with the addition of ultrasounds.

### 2.3. Bioactive Compounds’ Determination and Antioxidant Assays

**Figure 6:**
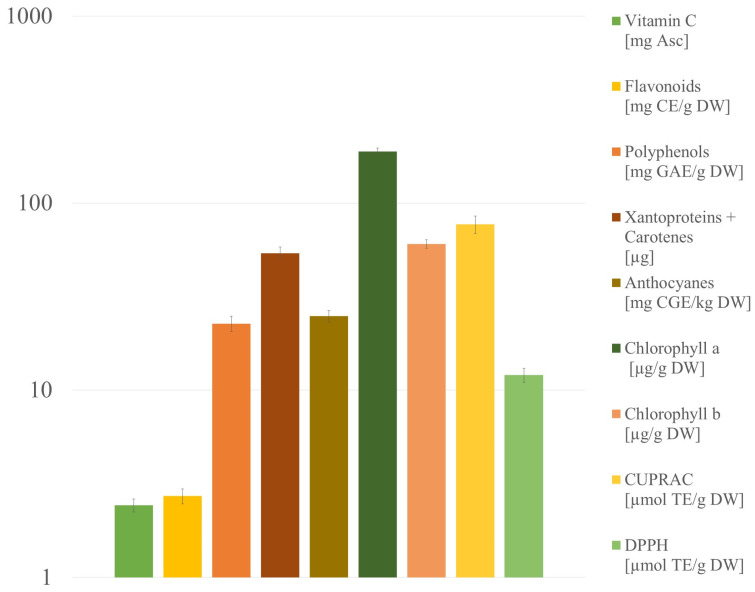
Bioactive compounds and antioxidant potential of kaffir lime peel.

Citrus fruits are a good source of phytochemical substances. The extraction of these bioactive compounds from citrus wastes can be performed using conventional extraction techniques (hydrodistillation, maceration, and solvent extraction) and via green extraction approaches (ultrasound-assisted extraction, enzyme-assisted extraction pulse electric field extraction, and microwave-assisted extraction) [44]. The presence of bioactive compounds in citrus waste makes them attractive owing to antimicrobial, anticancer, antidiabetic, antiplatelet aggregation, and anti-inflammatory activities, which is not without significance in the context of use as animal feed. The results of bioactive compound determination and antioxidant potential of kaffir lime peel are presented in Figure 6.

#### 2.3.1. Polyphenols

Polyphenols, as the products of plant metabolism, play a protective role against fungal and bacterial infections. They are an important component of the diet of both humans and animals, so their determination is crucial in the context of using peel wastes as animal feed or natural ingredients for cosmetics. The total polyphenols content (TPC) in kaffir lime was 22.63 ± 2.12 mg GAE/g DW. In our previous reports, the amount of total polyphenols extracted with methanol from kaffir lime juice was 15.79 ± 1.34 mg GAE/g DW [8]. The content of polyphenols in pomelo (*C. grandis)*, citron (*C. medica),* mandarin (*C. reticulata*), and grapefruit (*C. Sinensis*) was 14.93 ± 0.21, 8.88 ± 0.34, 23.46 ± 1.19, and 12.68 ± 0.39 mg GAE/g DW, respectively [45]. Kaffir lime peel is thus a better source of polyphenols than juice. Moreover, compared with the peel of other citrus fruits, the amount of total polyphenols in kaffir lime is relatively high.

#### 2.3.2. Chlorophylls

Another important group of bioactive compounds in kaffir lime is chlorophylls. They belong to the group of green natural pigments, commonly used in the medicine and food industries. Thanks to their antioxidant properties, they are used to prepare fortified and functional products. Commercially, chlorophylls are obtained from plants with green leaves, like spinach [46]. Such an approach requires special cultivation of plants for obtaining colorants, designed specifically for this purpose. Recent literature reports mention the use of citrus waste to obtain natural dyes that can be used in industry. The problem with obtaining pigments from natural raw materials is their high cost. The approach using citrus waste, therefore, seems to be economically and environmentally viable, especially while production would be performed concomitantly with the extraction of pectins or essential oils [47]. Among other citrus fruits, a high total chlorophyll (a + b) content was determined for different lime varieties (*Citrus aurantifolia* Swingle cv. ‘Paan’)—392.5 µg/g DW [48]. Kaffir lime may be a good source of chlorophylls of all citruses, especially owing to the high content of pectins that can be extracted concomitantly. Kaffir lime waste may be considered as functional animal feed because of its high chlorophyll content, which exerts prominent health benefits when consumed; that is, antioxidant antimutagenic, and antigenotoxic [49].

#### 2.3.3. Flavonoids

Flavonoids, as part of polyphenols, are able to interfere with the structure of the other compound and change its properties, from which their bioactive properties result. For example, they are anti-inflammatory agents because of the diminished formation of proinflammatory mediators (leukotrienes, prostaglandins, and reactive oxygen species) [50]. Citrus fruits, especially their peels, are rich dietary sources of flavonoids. Chatha et al. provided a determination of total polyphenols in citrus fruit peels [51]. Mussambi fruit peel extract contained the highest total flavonoid content (2.98 g CE/100 g DW) among all citruses tested. The results for other citruses were similar and ranged between 2.20 and 2.98 g CE/100 g DW. In our research, kaffir lime peel extract contained 2.72 mg CE/g DW. Thus, compared with other citruses, kaffir lime peel is an average source of flavonoids. However, when the peel is used as a waste animal feed, any content of these compounds adds to its value. It is worth mentioning that apple pomace, commonly used as animal feed, contained 1.19 ± 0.058 mg RE/g DW [52]. In this context, kaffir lime waste is a promising source of flavonoids.

#### 2.3.4. Anthocyanins

Anthocyanins, as part of flavonoids, show many bioactive functions. Research works showed that anthocyanin-rich dried fruits have positive effects on health-promoting markers in humans and other animals, and that is why they may find application in the poultry feed industry [53] Although citrus fruits are not the best sources of anthocyanins, while they are natural purple pigments, much research on this area is provided. Gorinstein et al. determined 4.5 ± 0.3 and 17.5 ± 1.5 mg CGE/kg DW in grapefruit juice and peel, respectively [54]. In our previous research, kaffir lime juice was analyzed and contained 63.45 ± 5.15 mg CGE/kg DW [8]. In turn, in this research, the anthocyanin content in kaffir lime peel content was determined to be 24.8 ± 1.8 mg CGE/kg DW..

#### 2.3.5. Carotenoids (Xanthophylls and Carotenes)

Carotenoids are the pigments responsible for the color of many citrus fruits. Citrus is a complex source of these compounds, with the biggest number of carotenoids found in any fruit [55]. Their content in flavedo depends on the maturity and thus the color of the fruit. Among the Citrus family, mandarin’s (*Citrus reticulata*) peel shows a high content of total carotenoids, that is, 2143 ± 25.24 μg/g DW [56]. Moreover, sweet orange peel is a good source of carotenoids, 31.57 ± 0.06 μg/g DW. [57]. *C. limon* (L.) Bur, commonly added to dishes, contains 110.0 ± 1.0 24 μg/g DW. Based on our research, it was stated that kaffir lime peel contains 53.86 ± 4.24 μg/g DW, which is a relatively high value among citrus [3].

#### 2.3.6. Vitamin C

Vitamin C, with a high antioxidant potential, is a common ingredient in cosmetic products. It is also necessary for the proper functioning of the body, so its content in animal feed as well as in diet supplements is highly recommended. Thanks to its antioxidant potential, it can be used topically in dermatology to treat and prevent changes associated with photoaging [58]. Citrus fruit juice is one of the best sources of vitamin C. Among all citruses, key lime peel is found to be a rich source of vitamin C (1.779 ± 0.78 mg Asc/g) [57]. Based on our research, kaffir lime peel showed a higher content of vitamin C, that is, 2.43 ± 0.19 mg Asc/g. Kaffir lime peel extract can be successfully used in cosmetics and drugs as a source of natural vitamin C.

#### 2.3.7. DPPH Free Radical Scavenging and CUPRAC Cupric Reducing Antioxidant Capacity

The antioxidant capacity of fruits is an important indicator of their in vitro potential as health promoters. The CUPRAC test assesses the ability of antioxidants in a sample to reduce Cu^2+^ to Cu^+^ in the presence of a chelating agent. The DPPH test is used to predict the activity of antioxidants through the mechanism by which they inhibit lipid oxidation, i.e., by scavenging DPPH radicals. The DPPH value for kaffir lime peels was 12.02 ± 1.02 µmol TE/g DW. CUPRAS assay results were 76.98 ± 8.1 µmol TE/g DW. *Citrus sinensis* (L.) Osbeck and *C. limon* DPPH values were 18.20 ± 1.62 and 23.07 µmol TE/g DW, respectively [19,59], so the kaffir peel lime antioxidant capacity is slightly lower than other citruses’ peels. The situation is slightly different in the case of the CUPRAC test, where the values for the kaffir lime peel were higher than for other citrus fruits’ peels (54.8 ± 2.34 µmol TE/g DW for *Citrus reticulata* and 52.31 µmol TE/g DW for *C. paradisi* [60,61]). These results provide an alternative way to make good use of kaffir lime peel to utilize it as a natural antioxidant source.

### 2.4. Micro and Macroelements

Chemical elements found in living organisms can be divided into microelements, macroelements, and ballast elements. Although the isolation of micro- and macroelements from citrus fruits has not been carried out so far, the current trends do not exclude this possibility, which is a promising prospect. According to Barros et al., citrus fruits are promising sources of mineral elements [62]. The presence of minerals in citrus waste makes it a good substrate for the production of bio-fertilizers. The results of the determination of selected elements in kaffir lime peel are presented in Table 6.

In the case of microelements, their importance lies in the regulation of the activity of enzymes, hormones, vitamins, and other factors determining the course of metabolic processes in organisms. In the samples of kaffir lime peel, it was possible to quantitatively determine five trace elements, i.e., iron, zinc, copper, manganese, and molybdenum. The remaining compounds in this group were below the limit of detection or quantification. Iron had the highest content, i.e., 32.72 ± 0.39 mg/kg DW. To compare, *Citrus maxima* peel extract contains 9.06 ± 0.79 mg/100 g [63]. Iron plays an important role in the supply of oxygen to the organs and muscles of living organisms. Although citrus is not the main dietary source of iron, its presence in fruit intended for animal feed is an added benefit. Second in terms of the content of kaffir lime in the peel, zinc is a recommended micronutrient in fertilizers for the production of corn [64]. The content of zinc was 16.09 ± 0.14 mg/kg DW. The high content of microelements is also an important aspect of the production of dietary supplements using plant materials. Therefore, kaffir lime peel is a good raw material for use in the food, agriculture, pharmaceutical, and cosmetic industries.

Macroelements are building materials for proteins, lipids, sugars, nucleotides, the skeletal system, and the external skeleton of animals. They have been widely used in the prevention and treatment of many diseases [65]. Among the macroelements, the most abundant elements in the peel extract were potassium and calcium, with a concentration of 10,820 ± 130 and 9416 ± 34 mg/kg DW, respectively. This is consistent with other studies. Dibanda Romelle et al. showed that orange and pomegranate peels had calcium as the most abundant mineral analyzed [66]. The high content was also determined in the case of sodium and magnesium, i.e., 1500 ± 28 and 1050.3 ± 7.3 mg/kg, respectively. To compare, *Citrus maxima* peel extract contains 46.12 ± 1.46 and 1.88 ± 0.05 mg/100 g, respectively [63]. The high content of these elements in kaffir lime citrus waste makes them good food for cattle. In turn, the high content of potassium is important in the context of bio-fertilizers, as potassium is one of the basic plant nutrients.

Ballast elements are chemical elements without a known function in metabolism [67] often harmful to humans, and often toxic at higher concentrations. Heavy metals are also among them. The elements are derived directly or indirectly from the polluted environment. In the kaffir lime peel, all of the ballast elements were found in trace amounts without particular importance for the use of citrus waste in the industry. In the case of aluminum and strontium, the concentrations were 77.46 ± 0.33 and 16.16 ± 0.22 mg/kg DW, respectively. Their oral exposure is usually not harmful, so their presence is not a limit while producing animal food from kaffir lime wastes. The presence of mercury and barium is undesirable, but the amounts indicated are relatively small.

## 3. Materials and Methods

### 3.1. Materials and Reagents

Kaffir lime fruits were bought in Thailand by the local distribution point in the Pomeranian Voivodship in December 2019 and transported to the laboratory in refrigeration conditions. Fruits for the analysis were taken from four batches, each composed of 70–80 pieces (about 3 kg). Fruits for analysis were washed with tap and distilled water and peeled manually. Fresh peel has undergone further preparation processes, such as drying, freeze-drying, mineralization, extraction, or hydrodistillation. D-(+)-galacturonic acid monohydrate, sulphamic acid, 3-phenylphenol, terpenes mix in methanol, Trolox (6-hydroxy-2,5,7,8,-tetramethyl-chroman-2-carboxylic acid), Folin–Ciocalteu reagent (FCR), gallic acid, 20-azobis-2-methyl-propanimidamide, FeCl_3_·6H_2_O, Folin Ciocalteu reagent (FCR), lanthanum (III) chloride heptahydrate, CuCl_2_·2H_2_O, 2,9-dimethyl-1,10-phenanthroline (neocuproine), 1,1-diphenyl-2- picrylhydrazyl (DPPH), potassium persulfate, and terpenes standards were purchased from Sigma-Aldrich (St. Luis, MO, USA). Methanol, citric acid, sodium hydroxide, and nitric acid were purchased from Avantor Performance Materials Poland S.A. K, Ba, Ca, Cd, Co, Cu, Zn, Ni, Pb, Pt, V, and Mo standards at a concentration of 1000 ± 2 mg/L; Mg standard solution at a concentration 1006 ± 4 mg/L; Fe standard at a concentration 1001 ± 2 mg/L; and Al standard at a concentration 998 ± 5 mg/L were obtained from Sigma-Aldrich (Darmstadt, Germany). Na standard at a concentration of 10,000 mg/L and Sr standard at a concentration of 1005 ± 5 mg/L in 4% HNO_3_ were purchased from MS Spectrum (Poland). Cr standard at a concentration of 1003 ± 3 μg/mL and Mn standard at a concentration of 1000 ± 6 μg/mL were purchased from CPI INTERNATIONAL (Santa Rosa, CA, USA). Nitric acid (65–70% purity) was obtained from Alfa Aestar (Regensburg, Germany). Mercury standard-MSHG at a concentration of 100.10 ± 0.43 μg mL^−1^ in 10% HCl was purchased from Inorganic Ventures, INC (Christiansburg, VA, USA). N-acetyl- L-cysteine was obtained from Sigma Aldrich (Germany). All other chemicals used in the study were of analytical grade.

### 3.2. Essential Oil Compositions

#### 3.2.1. Hydrodistillation

The essential oil was extracted from fresh kaffir lime peel by the hydrodistillation process. Here, 500 g of kaffir lime peel was crushed and extracted in glass distillation apparatus for 3 h and then separated in a laboratory glass separator. The extraction process was performed three times and the obtained products were combined. To enhance the yield of the extraction, a salt effect using NaCl treatment was performed. The essential oil was dried under anhydrous sodium. The yield of the oil obtained was calculated as a percentage. The essential oil was stored at 4 °C until further use.

#### 3.2.2. GS–MS Analysis

The GC–MS system (Shimadzu, Kyoto, Japan) consisted of a GCMS-QP2010Plus gas chromatograph mass spectrometer. Separation of the oil was performed using DB WAX 52 CB (Agilent Technologies, Santa Clara, CA, USA) chromatographic column (30 m × 0.25 mm id, film thickness 0.25 m). Hydrogen was used as the carrier gas at a constant flow rate of 24.1 mL/min. Injector and MS transfer line temperatures were set at 175 and 220 °C, respectively. The ion source temperature was 220 °C. The initial temperature was kept at 40 °C for 2 min and then gradually increased to 90 °C at a rate of 5 °C/min, then to 220 °C at a rate of 30 °C/min, and was held for 7 min. The total time of analysis was 23.23 min. The samples were injected into the GC–MS system in the split mode (split ratio of 15). The identification of the components was performed based on comparing retention times to the retention times of standards. The relative peak areas of each component in essential oil were calculated by normalization of the peak areas as the percentages of the total essential oil component. The results were all expressed as mean ± SD.

### 3.3. Pectins’ Characteristics

#### 3.3.1. Extraction

Acid extraction (AE) was used for the isolation of pectins from plant material. Fruits for pectin esterification were washed with tap and distilled water. Peel was manually separated for the pulp, cut into small pieces, and dried at 40 °C for 48 h. The dried peel was ground to a powder. For the extraction, 3 g of grounded power was weighed and 150 mL of distilled water was added (solid/liquid ratio 1:50). In order to select the best extraction conditions, an experiment determining the influence of two independent parameters, such as temperature (70–90 °C) and pH for nitric and citric acid on the extraction yield of kaffir lime pectin, was performed.

The pH of the solutions was adjusted to the desired values using citric and nitric acid. Samples were heated under cover at 70, 80, and 90 °C for 1 h. After heating, an equivalent amount of cooled methanol was added to precipitate the pectins and left for 24 h. The coagulated pectins were centrifuged at 10,000× *g* for 30 min and washed with methanol until the pH was neutral. Pectins were dried at 40 °C until a constant mass. For further use, pectins were powdered. In the case of ultrasound extraction (UAE), the same sample preparation and conditions as in acid extraction were applied with the addition of ultrasound treatment. Pectin yield was calculated as the ratio of the mass of pectin and the sample taken for extraction.

#### 3.3.2. Degree of Methylation

The degree of methylation (DM) of pectin was determined using the titrimetric method of Rodsamran [68]. Here, 0.1 g of dried pectin sample was moistened with 2 mL ethanol and dissolved in 20 mL water at 40 °C. After the pectin solution was completely dissolved, the sample was titrated with 0.1 M NaOH against phenolphthalein at the end-point (V_1_). Afterward, 10 mL of 0.5 M NaOH was added, the mixture was shaken vigorously, and then left for 20 min. After that, 10 mL of 0.5 M HCl was added and titrated with 0.1 M NaOH to a faint pink color that persisted after vigorous shaking (V_2_). The DM was calculated using the following equation:

(1) $$\mathrm{DM}\%=\frac{V_{1}}{V_{1}{+V}_{2}}\cdot 100\%$$

#### 3.3.3. Galacturonic Acid Content

Galacturonic acid content was determined based on the Melton method [69]. Here, 5 mg of dried pectin samples were hydrolyzed with 1 mL of concentrated sulfuric acid for 5 min, under constant stirring, and cooled in an ice bath, and the procedure was duplicated. Then, 0.5 mL of distilled water was added, the mixture was stirred for 5 min, then the next portion of water was added and it was mixed again for 5 min. The mixture was diluted to 10 mL with distilled water in volumetric flasks. The samples were separated in a centrifuge model MPW-352 (MPW MED. INSTRUMENTS, Warsaw, Poland) by centrifuging for 10 min at 2000× *g* at room temperature. The supernatant was used for the colorimetric assay as follows. To 400 μL of supernatant or standard (galacturonic acid in water), 40 μL of 4 M sulfamic acid/potassium sulfamate solution (pH = 1.6) was added and vortexed. Then, 2.4 mL of 75 mM sodium tetraborate/sulfuric acid solution was added. The mixture was heated (100 °C) for 20 min and then cooled in ice. Then, 80 µL m-hydroxydiphenyl solution was added to the sample and reagent control tubes. To the sample control, 80 μL 0.5% NaOH was added to determine the sugar coloring. Samples were then vortexed and the absorbance was measured after 10 min at 525 nm in a DR3900 Benchtop VIS Spectrophotometer with RFID Technology (Hach Company, Loveland, CO, USA) against the reagent control (a mixture of reagents from the above procedure without samples of pectins). The determination was performed in three repetitions.

#### 3.3.4. DPPH Free Radical Scavenging

The antioxidant potential of pectins was determined by the DPPH assay using the method previously reported in the literature [39]. In brief, 1 mL of pectin’s solutions of concentrations of 1, 3, 5, 7, 10, and 12 mg/mL were mixed with 0.2 mM DPPH methanol solution and incubated for 30 min at 24 °C in the dark. The absorbance was read at 517 nm using a Hach DR3900 spectrophotometer (Hach Company, Loveland, CO, United States) against methanol. The control was prepared by replacing the DPPH solution with anhydrous methanol.

#### 3.3.5. DSC Analysis

**Figure 7:**
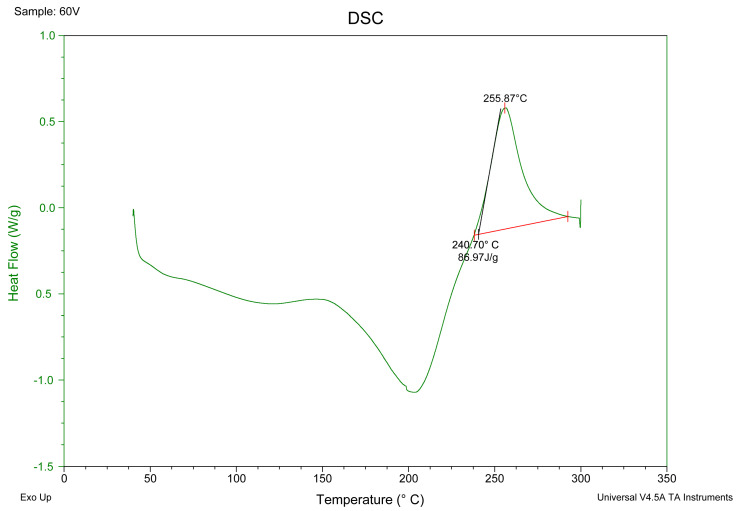
Calculation of the degradation temperature and enthalpy in DSC curves, based on 60U sample.

Differential scanning calorimetry (DSC Q20, TA Instruments-Waters LLC, New Castle, DE, USA) was used to investigate the thermal properties of the pectins according to the method previously described [35]. Then, 5 mg of dried pectin sample was added into a standard aluminum crucible and sealed by a non-hermetic seal. The crucible was heated from 40 °C to 300 °C at a heating rate of 10 °C/min in a dynamic inert nitrogen atmosphere (75 mL/min). Simultaneously, an empty standard aluminum crucible was used as a reference. Extrapolated peak temperature and enthalpy were calculated with the TA Universal Analysis software, as shown in Figure 7.

### 3.4. Bioactive Compounds’ Determination and Antioxidant Assays

Polyphenols were determined using the method described in our previous research with slight modifications [18]. The freeze-dried powders of investigated samples were immersed in methanol (1/10 *w/v*). The filtrate was collected three times with constant stirring of the mixture at every 24 h interval of a 72 h total collection period at room temperature. The extract was then concentrated under reduced pressure at 45 °C using a vacuum rotary evaporator. Then, lyophilized peel samples were determined by the Folin–Ciocalteu method. The absorbance was measured at 750 nm. The results were expressed as mg of gallic acid equivalents (GAEs) per g of dry weight (DW).

Flavonoids were determined by the method described by Papotuis [70]. Samples were extracted using methanol in ultrasounds for 20 min (vortexed every 5 min for 10 s) and centrifuged. Appropriate amounts of H_2_O, 5% NaNO_2_, 10% ACl_3_, and 4% NaOH were added and the absorbance was measured at 510 nm using the Hach DR3900 spectrophotometer (Hach Company, Loveland, Colorado, USA). The results were expressed as mg of catechin equivalents (CE) per g of the dry weight of the sample.

For anthocyanin determination, triple extraction with 15 mL of 80% methanol (pH = 2) for 10 min was performed. After each extraction, samples were centrifuged for 5 min and supernatants were collected. Then, 1 mL of combined extracts was filtered through a syringe filter. Absorbance was measured at 510 and 700 nm in buffers at pH 1.0 and 4.5 and calculated using the following formula: A = (A_520nm_ − A_700nm_)_pH1_._0_ − (A_520nm_ − A_700nm_)_pH4_._5_ with a molar extinction coefficient of cyanidin-3-glucoside of 29,600 and a molecular weight of 449.2 g/mol. The results were expressed as milligrams of cyanidin-3-glucoside equivalent (CGE) per g of DW.

Total carotenoids (xanthophyll + carotenes) were extracted with 100% acetone and determined spectrophotometrically at 454 nm, while chlorophylls were extracted with 85% acetone and determined spectrophotometrically at 660 and 642.5 nm [71]. The results were expressed in µg.

For the CUPRAC assay, 1 mL of copper (II)-neocuproine and NH4Ac buffer solution, extract of sample (or standard) solution, and H_2_O was added to a final volume of 4.1 mL. The absorbance at 450 nm was read against a reagent blank. The results were expressed as µmole Trolox/g DW.

Scavenging free radical potentials were analyzed in 3.9 mL of methanolic solution of 1,1-diphenyl-2-picrylhydrazyl (DPPH) with the samples of lime peel extracts in methanol (0.1 mL). The results were expressed as µmole Trolox/g DW [72].

Total ascorbic acid was determined by CUPRAC assay in water extract (20 mg/mL). The absorbance of the formed bis (Nc)-copper (I) chelate was measured at 450 nm [73].

### 3.5. Micro- and Macroelements’ Content

In order to determine the content of selected elements in the samples, they were subjected to microwave-assisted mineralization. For this purpose, about 1 g of the sample was weighed into a reaction vessel. Then, 8 mL of HNO_3_ was added to each reaction vessel. Mineralization proceeded for the first 20 min at 100 °C and the next 20 min at 180 °C. Then, the mineralized samples were placed in 25 mL flasks and supplemented with deionized water to the dash. After mixing, each solution was poured into stoppered plastic tubes. The samples prepared in this way were analyzed using atomic emission spectrometry. The 4210 MP—AES supplied by Agilent was used. Millipore—Milli-Q Water Purification System (USA) and Anton Paar Multiwave Go microwave mineralizer were used. Mercury/MA-3000 supplied by Nippon Instruments Corporation (NIC, Osaka, Japan) was used to analyze mercury using the cold vapor technique and purified dry air was used as the carrier gas.

Determinations were made at several wavelengths for each element. The final choice of the wavelength at which the determination was made was determined by the value of the coefficient R^2^ for the calibration curve. The results were expressed as mg/kg DW. Validation parameters are presented in Table 7.

### 3.6. Data Analysis

Microsoft^®^ Excel^®^ spreadsheet was used for data entry and calculations. The data were presented as a mean or mean ± SD (standard deviation) of at least three independent measurements. Where it was appropriate, one-way analysis of variance (ANOVA) was used to compare the mean values. A probability of 5% or less was accepted as statistically significant. Pearson’s correlation coefficient was used to determine correlations. The design of the experiment of pectins’ extractions was performed using STATISTICA 12 (StatSoft, Inc., Tulsa, OK, USA).

## 4. Conclusions

Kaffir lime peel, as a citrus waste, is a promising raw material in many branches of the industry thanks to its high content of valuable by-products, whose presence increases its dietary and therapeutic value. The pectin extraction experiment showed that citric acid is a good option for pectin extraction and that the yield of pectins increased with the temperature and lowered with an increase in pH. Kaffir lime peel is a good source of low methyl pectins that could be used in the food industry. According to the DSC results, increasing the temperature of extraction and the addition of ultrasounds have an influence on the structure of the obtained pectins. The main difference may be connected to the different amounts of methoxyl groups. Kaffir lime essential oil contains several terpenes with many bioactive properties and has a pleasant smell and is a good choice for aromatherapy and cosmetology. Because of the high content of bioactive compounds and minerals, kaffir lime peel can be used as animal feed or in the production of bio-fertilizers. In addition, thanks to its pro-health properties and pleasant smell, powdered kaffir lime peel can be an important component of dietary supplements in the form of capsules, as in the case of commercially available orange peel. The high levels of health-promoting compounds, compared with other citrus fruits, make the peel of kaffir lime worthy of consideration as a candidate for widespread use in dried or freeze-dried form. *Citrus hystrix* seems to have great value for utilization in many forms in the food, medical, cosmetic, or agriculture industries. The natural origin of this ingredient makes it even more attractive for these applications.

## Figures and Tables

**Table 1:** Chemical composition of kaffir lime peel essential oil.

No.	Chemical Compound *	Composition (%)
1	α-pinene	4.27
2	Camphene	0.17
3	Sabinene	31.9
4	β-pinene	26.3
5	Myrcene	0.81
6	p-cymene	0.79
7	β-phellandrene	9.34
8	Limonene	18.6
9	β-ocimene	0.27
10	Linalool	0.24
11	Citronellal	0.24
12	Terpinen-4-ol	0.23
13	α-Terpineol	0.33
14	Carveol	0.16
15	Citronellol	0.17

* Owing to the applied standard solution, particular terpene isomers were not distinguished.

**Table 2:** Yields of pectins obtained using different extraction conditions.

Extraction Conditions	Pectin Yield (%)
Acid extraction, 60 °C (60A)	7.6
Acid extraction, 70 °C (70A)	14.1
Acid extraction, 80 °C (80A)	17.6
Ultrasound acid extraction 60 °C (60U)	9.9
Ultrasound acid extraction, 70 °C (70U)	16.0
Ultrasound acid extraction, 80 °C (80U)	28.2

**Table 3:** The degree of esterification of kaffir lime pectins determined by the titration method.

Extraction Conditions	Degree of Methylation (%)
Acid extraction, 60 °C (60A)	3.45 ± 0.51
Acid extraction, 70 °C (70A)	2.81 ± 0.45
Acid extraction, 80 °C (80A)	4.00 ± 0.84
Ultrasound acid extraction 60 °C (60U)	3.81 ± 0.89
Ultrasound acid extraction, 70 °C (70U)	3.87 ± 0.03
Ultrasound acid extraction, 80 °C (80U)	2.91 ± 0.64

**Table 4:** The content of galacturonic acid in the samples of kaffir lime pectins depending on the extraction conditions used in terms of dry matter.

Extraction Conditions	Galacturonic Acid Content (mg GAL/g)
Acid extraction, 60 °C (60A)	650 ± 75
Acid extraction, 70 °C (70A)	503.2 ± 8.7
Acid extraction, 80 °C (80A)	439.4 ± 6.5
Ultrasound acid extraction 60 °C (60U)	312.8 ± 2.2
Ultrasound acid extraction, 70 °C (70U)	339.5 ± 1.7
Ultrasound acid extraction, 80 °C (80U)	367 ± 10

**Table 5:** Thermal properties of pectins determined by DSC.

	T_endo_ [°C]	ΔH_endo_ [J/g]	T_exo_ [°C]	ΔH_exo_ [J/g]
Acid extraction, 60 °C (60A)	203.40	334.6	256.21	54.91
Acid extraction, 60 °C (60U)	204.77	191.1	255.87	86.97
Acid extraction, 70 °C (70A)	204.15	320.5	253.49	70.49
Acid extraction, 70 °C (70U)	200.49	276.5	254.42	67.33
Acid extraction, 80 °C (80A)	204.26	151.5	256.82	65.52
Acid extraction, 80 °C (80U)	201.23	262.4	256.84	77.73

T_endo_, temperature of main endothermic peak (°C); ΔH_endo_, enthalpy of main endothermic peak (J/g); T_exo_, temperature of degradation (°C); ΔH_exo_, degradation enthalpy (J/g).

**Table 6:** Results of the determination of the content of selected elements in kaffir lime peel (x_m_ ± U, (k = 2)).

**Microelements Concentration ± U [mg/kg]**	
Fe	32.72 ± 0.39
Zn	16.09 ± 0.14
Cu	4.518 ± 0.039
Mn	7.008 ± 0.029
Co	<LOQ
Ni	<LOD
Cr	<LOQ
Mo	0.295 ± 0.017
V	<LOD
**Macroelements Concentration ± U [mg/kg]**	
Mg	1050.3 ± 7.3
Ca	9416 ± 34
K	10820 ± 130
Na	1500 ± 28
**Ballast Element Concentration ± U [mg/kg]**	
Cd	<LOD
Hg	0.0145 ± 0.0011
Pb	<LOD
Al.	77.46 ± 0.33
Ba	16.52 ± 0.29
Sr	16.16 ± 0.22
Pt	<LOD

**Table 7:** Validation parameters of the procedure for the determination of the selected elements in lime samples.

Analyte	WAVELENGTH [nm]	LOD [mg/kg]	LOQ [mg/kg]	Linearity					
				Calibration Range [mg/kg]					
				Min.	Max.	Points	Rep.	Calibration Curve	R^2^
Microelements									
Fe	371.993	0.33	1.0	1.0	100	8	4	y = 5510x − 1049	0.9997
Zn	213.857	0.19	0.58	0.58	10	9	4	y = 12014x + 96	0.9995
Cu	327.395	0.026	0.077	0.30	20	6	4	y = 44555x − 1626	0.9999
Mn	403.076	0.0064	0.019	0.019	1.0	5	4	y = 28990x + 44	0.9999
Co	345.351	0.012	0.035	0.050	1.0	5	4	y = 13331x − 2.4	0.9999
Ni	361.939	0.0070	0.021	0.10	20	7	4	y = 5637x − 338	0.9999
Cr	425.433	0.0027	0.0082	0.01	10	8	4	y = 29402x + 29	0.9999
Mo	386.410	0.0060	0.018	0.018	20	9	4	y = 15860x + 29	0.9995
V	437.923	0.0057	0.017	0.017	20	9	4	y = 7795x + 42	0.9997
Macroelements									
Mg	279.553	0.40	1.2	1.2	40	6	4	y = 152325x + 37340	0.9996
Ca	430.253	2.0	6.0	10	250	6	4	y = 898.5x + 1376	0.9995
K	766.491	0.16	0.48	2.5	20	4	4	y = 48347x − 16941	0.9997
Na	568.263	1.1	3.3	10	200	5	4	y = 34.5x − 34.1	0.9998
Ballast substances									
Cd	228.802	0.022	0.066	0.066	20	8	4	y = 23459x − 525	0.9998
Hg	253.700	0.00096	0.0029	0.0029	0.10	10	3	y = 1.001x − 0.15	0.9999
Pb	405.781	0.012	0.035	0.050	5.0	6	4	y = 2776x − 77	0.9999
Al	396.152	0.088	0.26	1.0	100	8	4	y = 20008x + 632	0.9998
Ba	493.408	0.21	0.63	0.63	3.0	4	4	y = 20318x + 5708	0.9962
Sr	421.552	0.0045	0.013	0.013	40	6	4	y = 29277x + 58	1.0000
Pt	265.945	0.075	0.23	0.40	4.0	4	4	y = 3628x + 499	0.9994

## Data Availability

The data presented in this study are available upon request from the corresponding author. The data are not publicly available for privacy reasons.

## Sample Availability

Samples of the compounds are not available from the authors.
